# Piloting a surveillance system to monitor the global patterns of drug efficacy and the emergence of anthelmintic resistance in soil-transmitted helminth control programs: a Starworms study protocol

**DOI:** 10.12688/gatesopenres.13115.1

**Published:** 2020-03-10

**Authors:** Johnny Vlaminck, Piet Cools, Marco Albonico, Shaali Ame, Thipphavanh Chanthapaseuth, Vanisaveth Viengxay, Dung Do Trung, Mike Y. Osei-Atweneboana, Elias Asuming-Brempong, Mohammad Jahirul Karim, Abdullah Al Kawsar, Jennifer Keiser, Virak Khieu, Babacar Faye, Innocent Turate, Jean Bosco Mbonigaba, Nadine Ruijeni, Eliah Shema, Ana Luciañez, Ruben Santiago Nicholls, Mohamed Jamsheed, Alexei Mikhailova, Antonio Montresor, Denise Mupfasoni, Aya Yajima, Pauline Ngina Mwinzi, John Gilleard, Roger K. Prichard, Jaco J. Verweij, Jozef Vercruysse, Bruno Levecke

**Affiliations:** 1 Department of Virology, Parasitology and Immunology, Ghent University, Merelbeke, Belgium; 2 Center for Tropical Diseases, Sacro Cuore Don Calabria Hospital, Negrar, Italy; 3 Department of Life Sciences and Systems Biology, University of Turin, Turin, Italy; 4 Laboratory of Parasitology, Public Health Laboratory Ivo de Carneri, Chake Chake, Tanzania; 5 Vientiane Office, World Health Organization, Vientiane, Lao People's Democratic Republic; 6 National institute of Health, Ministry of Health, Vientiane, Lao People's Democratic Republic; 7 National Institute of Malariology, Parasitology and Entomology, Ministry of Health, Hanoi, Vietnam; 8 Biomedical and Public Health Research Unit, CSIR-Water Research Institute, Accra, Ghana; 9 Filariasis Elimination, STH Control and Little Doctor Programme, CDC, Directorate General of Health Services, Dhaka, Bangladesh; 10 Department of Medical Parasitology and Infection Biology, Swiss Tropical and Public Health Institute, Basel, Switzerland; 11 National Centre for Parasitology, Entomology and Malaria Control, Ministry of Health, Phnom Penh, Cambodia; 12 Service of Parasitology and Mycology, University of Cheikh Anta DIOP, Dakar, Senegal; 13 The institute of HIV/AIDS Disease Prevention and Control, Rwanda Biomedical Center, Kigali, Rwanda; 14 College of Medicine and Health Sciences, University of Rwanda, Kigali, Rwanda; 15 Neglected Tropical Diseases, Communicable Diseases and Environmental Determinants of Health, Pan American Health Organization, Washington D.C., USA; 16 Department of Communicable Diseases, World Health Organization, New Delhi, India; 17 Department of Control of Neglected Tropical Diseases, World Health organization, Geneva, Switzerland; 18 Western Pacific Regional Office, World Health Organization, Manilla, Philippines; 19 Expanded Special Project for Elimination of NTDs (ESPEN), World Health Organization, Brazzaville, Congo; 20 Department of Comparative Biology and Experimental Medicine, University of Calgary, Calgary, Canada; 21 Institute of Parasitology, McGill University, Montreal, Canada; 22 Laboratory for Parasitology, Elisabeth-Tweesteden Hospital, Tilburg, The Netherlands

**Keywords:** Soil-transmitted helminths, Kato-Katz thick smear, egg reduction rate, preventive chemotherapy, benzimidazoles, anthelmintic drug resistance, single nucleotide polymorphisms, next generation sequencing, loop-mediated isothermal amplification

## Abstract

To eliminate soil-transmitted helminth (STH) infections as a public health problem, the administration of benzimidazole (BZ) drugs to children has recently intensified. But, as drug pressure increases, the development of anthelmintic drug resistance (AR) becomes a major concern. Currently, there is no global surveillance system to monitor drug efficacy and the emergence of AR. Consequently, it is unclear what the current efficacy of the used drugs is and whether AR is already present. The aim of this study is to pilot a global surveillance system to assess anthelmintic drug efficacy and the emergence of AR in STH control programs. For this, we will incorporate drug efficacy trials into national STH control programs of eight countries (Bangladesh, Cambodia, Lao PDR, Vietnam, Ghana, Rwanda, Senegal and a yet to be defined country in the Americas). In each country, one trial will be performed in one program implementation unit to assess the efficacy of BZ drugs against STHs in school-aged children by faecal egg count reduction test. Stool samples will be collected before and after treatment with BZs for Kato-Katz analysis and preserved to purify parasite DNA. The presence and frequency of known single nucleotide polymorphisms (SNPs) in the β-tubulin genes of the different STHs will subsequently be assessed. This study will provide a global pattern of drug efficacy and emergence of AR in STH control programs. The results will provide complementary insights on the validity of known SNPs in the ß-tubulin gene as a marker for AR in human STHs as well as information on the technical and financial resources required to set up a surveillance system. Finally, the collected stool samples will be an important resource to validate different molecular technologies for the detection of AR markers or to identify novel potential molecular markers associated with AR in STH.

## Introduction

Today, preventive chemotherapy (PC) is the main strategy to control the morbidity that is caused by soil-transmitted helminths (STHs), which include ( *Ascaris lumbricoides*, *Trichuris trichiura*, *Necator americanus* and *Ancylostoma duodenale*). For this, a single, oral dose of albendazole (ALB; 400 mg) or mebendazole (MEB; 500 mg) is periodically administered to at-risk populations (i.e., preschool-aged (preSAC), school-aged children (SAC) and women of reproductive age) ^1^. The treatment coverage of SAC in control programs has increased from approximately 30% in 2010 to 69% in 2017 ^2^. The ultimate goal is to reach a 75% coverage in target populations and to push towards an elimination of the public health problems associated with moderate-to-heavy intensity STH infections ^3^. As of 2017, 21 countries have received PC STH coverage ≥75% for 5 years or longer and seven countries have eliminated STH as a public health problem ^4^.

The ever-increasing amount of anthelmintic treatments provided to the population could eventually give rise to the development of anthelmintic resistance (AR), as it has occurred in veterinary medicine ^5– 7^, where AR was detected in helminth populations within a decade following the introduction of any anthelmintic class ^8^. First, the two drugs provided in STH control programs (ALB and MEB) are both from the same drug class (benzimidazoles; BZs) and have an identical mode of action (preventing the polymerization of microtubules). Thus, if AR would arise against one of these drugs, it would likely also affect the efficacy of the other BZ drug. Second, it is important to note that BZ drugs are administered in single doses, a practice that never achieves 100% efficacy ^9– 12^. Although operationally justified, these single-dose drug regimens may further support the development of AR. Finally, there are very few alternative drugs that are licensed for the treatment of STH infections in humans ^13, 14^.

There is thus an urgent need to design a global surveillance system that is able to detect diminishing drug efficacy due to the development of AR in STHs. In parallel, drugs of different anthelmintic classes and modes of action should become more accessible, and the discovery and development of novel treatments stimulated.

Currently, there is no global surveillance system to monitor the efficacy of anthelmintic drugs or the emergence of AR. As a result, we have an inadequate understanding of the current efficacy of the administered drugs. There are some important obstacles to address that complicate the implementation of such a global surveillance system. First, there are only a limited number of laboratories and staff with sufficient experience to perform drug efficacy surveys and to analyse and report the data. Second, there is no quality assurance system in place to guarantee the accuracy of the obtained data. Third, at this time, there are no validated markers linked to AR in human STHs or diagnostic methods that allow for an early, on-site detection of AR. Finally, there is an overall lack of guidance on how to design surveys to monitor drug efficacy within PC programs.

In 2016, the Bill and Melinda Gates Foundation funded the Starworms project (Stop Anthelmintic Resistant Worms; www.starworms.org) ^15^. The main objective of this project is to strengthen the monitoring and surveillance of drug efficacy and AR in STH control programs. In a first work package of the Starworms project, we evaluated different diagnostic methods to measure drug efficacy and further investigated the presence and distribution of AR-related single nucleotide polymorphisms in STHs ^16, 17^. The current protocol describes the objectives and approach for the second work package of the Starworms project, and briefly discusses the expected output.

The overall aim of the studies performed as part of the second Starworms work package is to pilot a surveillance system to assess anthelmintic drug efficacy and the emergence of AR in eight countries were PC coverage has been high for at least five years.

The specific objectives are to:

1. Assess the prevalence of moderate-to-heavy intensity infections of the different STHs.
2. Assess the drug efficacy of a single dose of BZ drugs against STH infections.
3. Assess the frequency of the ß-tubulin single nucleotide polymorphisms (SNPs) linked to BZ resistance.
4. Identify implementation-related barriers and opportunities for monitoring drug efficacy and AR in national STH control programs.
5. Expand the Starworms repository of STH samples.

## Protocol

### Field trial design

A drug efficacy trial will be performed in eight STH-endemic countries during their national PC program. These trials were registered on the 22 ^nd^ of November 2019 on Clinicaltrials.gov (ID: NCT04177654; https://clinicaltrials.gov/ct2/show/NCT04177654). The study will focus on the target population of the country’s STH control programs, namely SAC (age 5–14). At the start of the trial (baseline), SAC will be required to provide a fresh stool sample. Children that meet all inclusion criteria and none of the exclusion criteria ( Table 1) will be enrolled in the study and will receive a study-specific identifier. Under direct supervision, each participant will be treated with a single oral dose of BZ drug. The choice of BZ drug will depend on the drug used in the STH control program ( Table 2). The drug used in the study will be provided by the national PC program. The duplicate Kato-Katz thick smear method will be used to determine the fecal egg counts (FECs; expressed in eggs per gram of stool (EPG)) for each STH in the stool samples provided by the children. Two to three weeks post drug administration, a second stool sample will be collected from each child that tested positive for any STH during baseline screening. These stool samples will again be examined by duplicate Kato-Katz. Children who remained positive for any STH following treatment will receive a second treatment with a BZ drug. Baseline samples that are positive for any STH as well as all follow-up samples will be preserved for molecular analysis (see below). The different steps of the trials are shown in Figure 1.

**Table 1.** Inclusion and exclusion criteria endorsed during the recruitment of participants for the field trials.

Inclusion criteria	Exclusion criteria
• Participant, male or female, is 5–14 years of age • Participant is in healthy condition (based on physical examination and medical history) • Parent(s)/guardians of participant signed an informed consent document indicating that they understand the purpose of, and procedures required for the study and that they allow their child to participate in the study • Participant of ≥6 years old has assented to participate • Participant of ≥12 years old has signed an informed consent document indicating that they understood the purpose of the study and the procedures required for the study and that they want to participate in the study • Participant provided a stool sample of minimum 5 grams at baseline and follow-up	• Participant has active diarrhoea (defined as the passage of 3 or more loose or liquid stools per day) at baseline or follow-up • Participant is experiencing a severe concurrent medical condition or has an acute medical condition • Participant has a known hypersensitivity to benzimidazole drugs • Participant received anthelmintic treatment within 90 days prior to baseline sample collection • Participant vomited within 4 hours following drug ingestion • Participant has not swallowed the entire tablet

**Table 2.** Overview of countries selected to monitor drug efficacy and the emergence of anthelmintic resistance. The median coverage between 2012 and 2016, the benzimidazole (BZ) drug administered in the soil-transmitted helminth (STH) control program, the frequency of preventive chemotherapy (PC) and the presence of other neglected tropical disease (NTD) programs (schistosomiasis and lymphatic filariasis (LF)) for the selected countries. SAC: school-aged children; DEC: diethylcarbamazine; IVM: ivermectin; PZQ: praziquantel.

WHO Region / Country	Median national coverage * of SAC (%)	Year from when ≥ 50% PC coverage in SAC was reported	BZ drug used	Number of rounds of PC with BZ/year	Other NTD programs (drug(s) used)
**Africa**					
Ghana	59.0	2009	ALB	1 – 2	Schistosomiasis (PZQ) LF (IVM + ALB)
Rwanda	98.6	2008	ALB	1 – 2	Schistosomiasis (PZQ)
Senegal	60.0	2012	ALB	1	Schistosomiasis (PZQ) LF (IVM + ALB)
**The Americas ^$#^**					
Nicaragua	100	2013	MEB	1	_
**South-East Asia**					
Bangladesh	86.4	2012	MEB/ALB	2	LF (DEC + ALB)
**Western Pacific**					
Cambodia	95.1	2003	MEB	2	_
Lao PDR	86.9	2006	MEB/ALB	1 – 2	LF (DEC + ALB)
Vietnam	76.9	2006	MEB/ALB	1 – 2	_

* National coverage = proportion of the SAC population requiring PC for STH in the country that have been treated.

$ Nicaragua has not yet confirmed their participation in the study. Through collaboration with the Pan American Health Organization we hope to receive their confirmation shortly or to identify another potential participating country if the response was negative.

# At the time of submission of the study protocol to the ClinicalTrials.gov database (NCT04177654) Haiti was still included as a possible country from the Americas.

**Figure 1.**
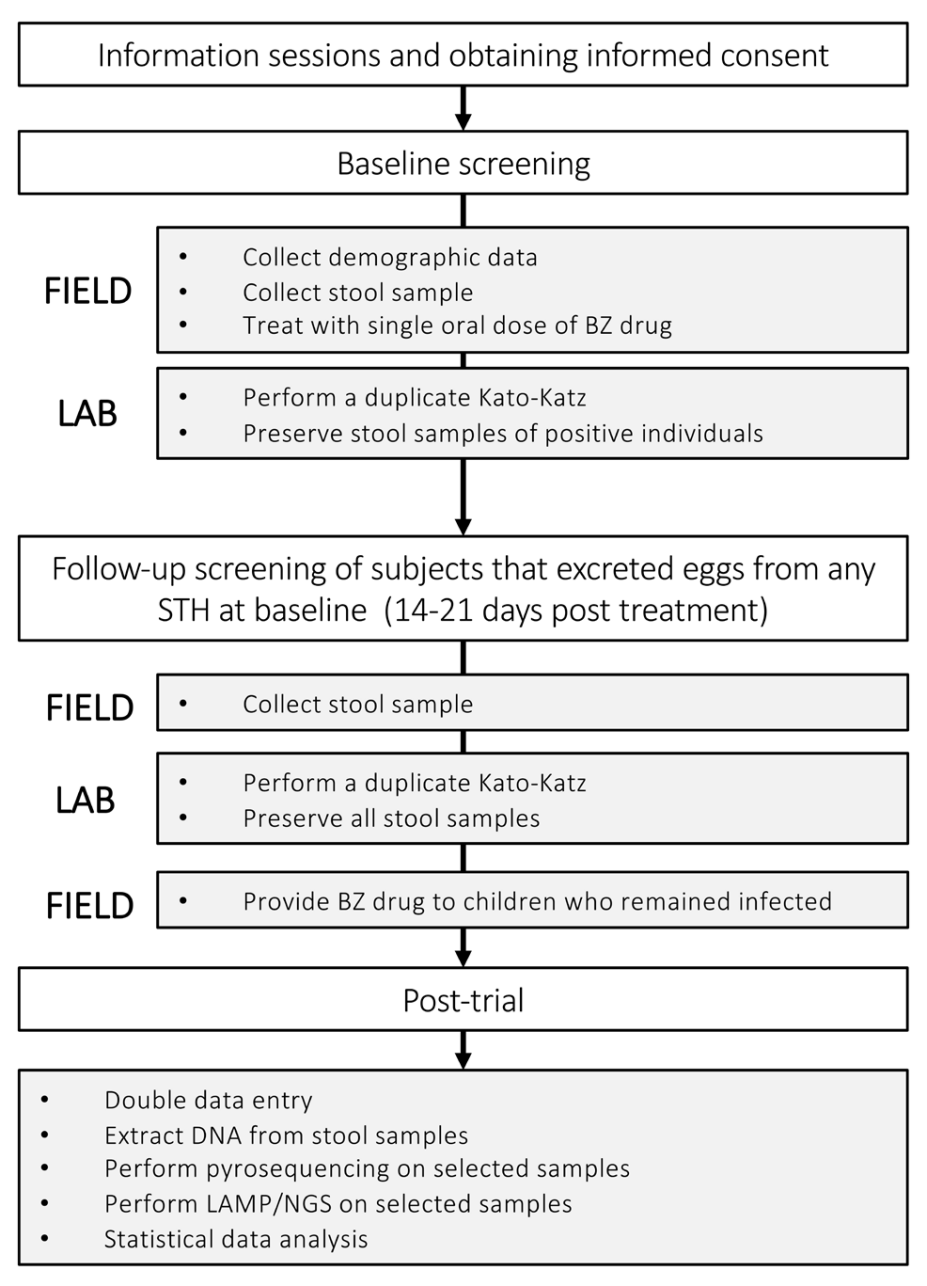
An overview of the different consecutive steps of the drug efficacy trials.

### Study site selection

Study sites were selected in two consecutive steps. First, countries were selected, after which potential study sites within each country were identified. The selection of the countries was based on the Preventive Chemotherapy and Transmission Control (PCT) databank for soil-transmitted helminthiasis, accessed in Nov 2017. This databank documents the number of preSAC and SAC requiring PC and the coverage for these at-risk populations for each country. We selected countries that:

- are part of WHO African Region (AFRO), Region of the Americas (AMR), South-East Asia Region (SEAR) or Western Pacific Region (WPR).
- reported national PC coverage data for the last 5 years (from 2012 to 2016)
- had a median national PC coverage for SAC of at least 50%
- reported subnational coverage data to WHO HQ for the last 4 years (from 2013 to 2017)

The cut-off for the median national PC coverage was set arbitrarily at 50%. This cut-off allows the inclusion of countries (i) where PC coverage was increasing over time and (ii) where PC coverage was high in some areas, but low in other areas. The selection on the availability of subnational data was made at WHO headquarters (by AMi). From the 138 countries included in the PCT data bank for STH, 13 countries met the aforementioned criteria. In collaboration with WHO regional offices, a subset of eight countries were selected from these 13 countries. For each of these countries, the median coverage of SAC between 2012 and 2016, the BZ drugs administered, the number of rounds of PC of BZ drugs and the presence of other neglected tropical disease (NTD) programs (schistosomiasis and lymphatic filariasis) is reported in Table 2.

Within these selected countries, we further identified geographical areas to which decisions on implementation of MDA apply (implementation units; IUs). We selected those IUs where: (i) subnational coverage data on SAC was reported between 2013 and 2016 and (ii) the minimum cumulative coverage of SAC over this period was at least 90%. The cumulative coverage equals to the sum of coverage of each round of PC. Coverage was calculated by dividing the number of SAC covered in a particular round of PC over the total number of SAC in that IU.

In the case that more than 10 IUs met the aforementioned criteria, and that there were no obvious differences that allowed to further prioritize IUs (e.g. total number of rounds over the 4-year period and presence of schistosomiasis/lymphatic filariasis program), the units where ordered according to decreasing coverage and the top 10 IUs were selected. Table 3 reports per country, the total number of IUs for which sub-national data was available, the administrative level at which the PC is administered and the number of units withheld based on the selection criteria. A list of the IUs is provided in *Extended data:* S1. The final selection of the IU where the study will be performed is done in close collaboration with country representatives and will depend on elements such as the availability of recent coverage and STH prevalence data, the presence of sentinel schools and other practical considerations (e.g. laboratory facilities, accessibility of the schools, etc.).

**Table 3.** The number of implementation units available and withheld for the eight selected countries. This table indicates the administrative level at which the PC is administered, the total number of implementation units (IUs) for which sub-national data was available, and the number of IUs withheld for each selected country. PC: preventive chemotherapy; PZQ: praziquantel.

WHO Region / Country	Administrative level of IU	Number of IUs	Number of IUs withheld
**Africa**			
Ghana	District	63	3
Rwanda ^a^	District	31	10
Senegal	Department	67	10
**The Americas**			
Nicaragua ^b^	Department	19	10
**South-East Asia**			
Bangladesh ^c^	District	64	10
**Western Pacific**			
Cambodia ^b^	Province	13	10
Lao PDR	Province	18	10
Vietnam ^d^	Province	63	9

^a^ the presence of schistosomiasis program was also considered - IUs reported at least two rounds of PZQ over the 4 years; ^b^ the IU representing the 10 highest minimum coverage; ^c^ the IU representing the 9 highest minimum coverage + 1 IU where a community based program was implemented in at least one year; ^d^ the number of multiple rounds per year was also considered – IUs reported multiple rounds in every year

### Sample size calculation

A sample size was calculated that allows for correctly identifying IUs where the efficacy of BZ drugs (ALB / MEB) against STHs is ‘reduced’, ‘doubtful’ and ‘satisfactory’ with a probability of at least 95%. The WHO criteria to classify BZ drugs into ‘reduced’, ‘doubtful’ and ‘satisfactory’ are summarized in Table 4. Given the differences in criteria across both the STH species and BZ drugs, a separate sample size was calculated for each STH species and for each BZ drug. To calculate the sample sizes, a simulation study was performed. This simulation study considered variation (i) in egg reduction rates (ERR) across STH species and participants, (ii) in baseline FECs across and within STH species, and (iii) variation in FECs introduced by the egg counting process. Based on the simulation, at least 150, 140 and 95 complete cases (i.e., participants positive at baseline and for which a follow-up sample was also collected and screened) are required for *Trichuris*, hookworm and *Ascaris*, respectively. If a child is positive for more than one STH species at baseline it thus serves as a case for each of the respective STH species. *Extended data:* S2 provides a more detailed description of the sample size calculation.

Given that the prevalence of STHs can vary significantly across schools within one IU, a number of schools per IU will be sampled. This number of schools corresponds with the number of sub-administrative levels within the selected IU. Per school, a random sample of a minimum of 100 participants will be enrolled.

### Laboratory procedures

***Egg counting.*** Following collection, stool samples will be homogenized and an aliquot will be used to prepare two Kato-Katz thick smears (standard operating procedure (SOP) in *Extended data:* S3). For each stool sample, two Kato-Katz thick smears will be prepared. Within 30–60 min after preparation of the slides, the presence of STH eggs is evaluated. The number of *A. lumbricoides*, *T. trichiura* and hookworm eggs counted per slide will be recorded. The two slides from the same sample will be examined by two different technicians.

A senior researcher, who is blinded to the initial FECs, will randomly selected a subset of samples (10% of total number of samples) to re-evaluate. In case of discrepancies, a third researcher will recount the STH eggs. Discrepancies will be defined as (i) false negatives/positives, (ii) difference in egg counts >10 when the total number of eggs counted ≤100 or (iii) difference in egg counts >20% when more than 100 eggs are counted ^19^. *Extended data:* S4 provides the SOP regarding the quality control of the egg counting.

***BZ resistance-associated molecular markers.*** After performing the duplicate Kato-Katz, stool samples aliquots will be preserved and stored for future molecular analysis. For each sample, a minimum of 2 g of stool will be preserved in an equal volume of 100% ethanol. At baseline, samples will be preserved if they are positive for at least one STH species. During follow-up, every sample will be preserved, irrespective of the STH egg counts. *Extended data*: S5 provides the SOP on stool sample preservation. The preserved stool samples will be stored at room temperature in sample boxes. Material will be shipped to Belgium for DNA extraction and molecular analysis. First, a mechanical lysis step including bead beating is used to break open the STH eggs and free the parasite DNA. After this, the automated QiaSymphony platform will purify the DNA from the lysate to maximize the yield of STH DNA obtained from the stool sample ^20^. The mechanical lysis step will be performed at Ghent University (Belgium) and the DNA purification at the Elizabeth-Tweesteden Hospital (The Netherlands) (SOP in *Extended data:* S6). Presence and quantity of STH DNA will be assessed Elizabeth-Tweesteden hospital by means of qPCR as described previously ^16, 20^

We will also assess the presence of genetic markers associated with BZ resistance using a variety of molecular tools. A Loop-mediated isothermal amplification (LAMP) assay and pyrosequencing will be performed at McGill University, Canada ^21, 22^. A deep amplicon sequencing approach will be tested by project collaborators at Calgary University (Canada) ^23^ and a novel digital droplet PCR for the detection of β-tubulin gene SNPs developed at Ghent University will also be applied.

Selected stool and DNA samples will be stored at Ghent University (WHO Collaborating Centre on Monitoring Drug Efficacy against soil-transmitted helminthiasis), and will be included in the Virtual STH Sample Inventory (VSSI) of the Starworms project. This sample repository will form a basis for validating novel molecular tools and identifying AR resistance mechanisms/markers other than the SNPs in genes encoding for the β-tubulins. They will also serve as historic reference samples for these IUs.

### Identifying implementation-related barriers and opportunities to monitor drug efficacy and AR in national control programs

By embedding eight drug efficacy studies within the national control programs worldwide, we will gain insights into potential barriers and opportunities that are related to the implementation of drug efficacy monitoring in national control programs. To do so we will (i) estimate the costs (both financial and in terms of human resources) to incorporate the monitoring of drug efficacy and AR in the national program for each of the selected countries ( *Extended data:* S7), (ii) organize focal group discussions with key persons in the national program associated to identify opportunities and barriers, (iii) field-test and evaluate the ParaDrug tool for the automated analysis of drug efficacy trials.

### Data management

The data that will be collected during the studies will be recorded in study-specific record forms ( *Extended data:* S8). The original data that is captured in the field will be stored on these paper documents. Later, these documents will be scanned and stored digitally. The raw data will be entered independently by two data entry clerks into study-specific Excel files ( *Extended data:* S9; double data entry). Following the double data entry, the files of both data entry clerks are evaluated for possible discrepancies (SOP in *Extended data:* S10). If mismatches are identified, true values will be verified by checking the original record form or its digital copy.

### Study coordination and management

Institutional review board (IRB) approval for these studies will be obtained from Ghent University and from each individual site. The local principal investigator (PI) is responsible to perform the trial procedures according to the original study protocol and using provided SOPs. The local project PI and the Starworms team at Ghent University will work together to coordinate the study. A member of the Starworms team will visit the study site prior to the start of the efficacy trial to inform local team members on the study design, to familiarise them with the different study documents and to provide both theoretical and practical training on the different laboratory techniques that will be used during the trial.

### Statistical data analysis

Following duplicated data entry and quality control, the data will be stored in a final, protected dataset to be used for statistical analysis. At the end of the project, and after finishing and publishing the data, this dataset will be published on the project website ( www.starworms.org). All statistical analysis will be performed in R ^24^. Levels of significance will be set at *p* <0.05.

***Assessing infection prevalence and intensities.*** Samples collected at baseline that contain helminth eggs will be classified into low, moderate, or heavy intensity infection based on the thresholds proposed by WHO ^25^. The proportion of moderate-to-heavy infection intensities will be determined separately for each STH species and study site.

***Assessment of the drug efficacy.*** The efficacy of a single, oral dose of BZ drug will be calculated and reported separately for each of the STHs ( *Ascaris*, *Trichuris* and hookworms), using the following formula:

$$\begin{array}{ll} \text{ERR}=100\%\times & \frac{\text{(arithmetic mean (FEC at baseline) - arithmetic mean (FEC at follow-up))}}{\text{arithmetic mean (FEC at baseline)}} \end{array}$$

The corresponding 95% confidence intervals (95% CI) will be calculated as described by Levecke *et al.*, 2018 ^26^. The drug efficacy classification will be based on the WHO criteria ( Table 4).

**Table 4.** Criteria to classify benzimidazole drug efficacy against soil-transmitted helminths ^18^. ERR: egg reduction rates.

Drug		Reduced	Doubtful	Satisfactory
Single, oral dose of 400 mg albendazole				
	*Ascaris*	ERR ≤ 85%	85% < ERR < 95%	ERR ≥ 95%
	*Trichuris*	ERR ≤ 40%	40% < ERR < 50%	ERR ≥ 50%
	Hookworms	ERR ≤ 80%	80% < ERR < 90%	ERR ≥ 90%
Single, oral dose of 500 mg mebendazole				
	*Ascaris*	ERR ≤ 85%	85% < ERR < 95%	ERR ≥ 95%
	*Trichuris*	ERR ≤ 40%	40% < ERR < 50%	ERR ≥ 50%
	Hookworms	ERR ≤ 60%	60% < ERR < 70%	ERR ≥ 70%

### Ethical approval and consent to participate

The IRB of the Faculty of Medicine of Ghent University (Belgium) has reviewed and approved the study protocol (Ref. No B670201837218). The institutional review boards associated with each trial site will also review the study protocol. At the start of each trial, the local PI and a team of field officers will visit the schools that will be included in the study. School directors, teachers, children and if possible, the parents will be informed on the planned trial and sampling methods. The written consent form will be prepared in the local language and provided to the children’s parents or guardians. Only children (i) who are willing to participate and (ii) whose parents or guardians have signed the informed consent form will be included in the study. For children older than 12, an additional, separate written informed consent form will be provided. The used consent forms are provided as part of a summarized protocol in English, French and Spanish ( *Extended data:* S11–13). Material transfer agreements between the leading local organization and Ghent University will govern the transfer of collected samples.

### Dissemination of study results

The study results obtained from the different drug efficacy trials will be combined and published in peer-reviewed scientific journals. Drug efficacy reports will be prepared per country and shared with key stake holders including country program managers and WHO representatives at the local and international level. Study data will be published as supplementary information to the manuscripts describing said results as on our project website ( www.starworms.org).

## Study status

Recruiting.

## Discussion/conclusions

To our knowledge, this is the first time a large-scale, multicentre study will document global patterns of anthelmintic drug efficacy and the emergence of AR applying standardized methodologies. We applied a rigorous selection approach performed in close collaboration with WHO headquarters, the different representatives from each WHO region and national program managers. This not only allowed for a transparent and strategic identification of countries and areas with an elevated risk to develop AR, it also envisioned that this approach is essential to strengthen the capacity within the national programs to independently continue monitoring of drug efficacy and the emergence of AR.

It is expected that the results will provide important information on (i) the current STH prevalence and infection intensity after multiple rounds of intense PC; (ii) global patters of efficacy of BZ drug administered in the control program; and (iii) the presence of genetic markers associated with BZ resistance. Besides the research findings, this study will also form an important resource for countries that want to establish their own surveillance system and for future research on the molecular detection of AR. For example, on top of the general selection framework, the different SOPs, it also includes a brief summary of the protocol and informed consent forms, that can be further customized to national requirements prior submission for ethical approval. To enhance the use of these documents, the documents are made available in English, French and Spanish ( *Extended data:* S11–13). Finally, the collected and stored stool samples will allow validation of future molecular technologies for the detection of AR or to identify novel potential molecular markers associated with AR in STH.

## Data availability

### Underlying data

No data are associated with this article. ****

### Extended data

Open Science Framework: StarwormsWP02, https://doi.org/10.17605/OSF.IO/M6VN7 ^27^.

This project contains the following extended data:

- **S1**: Program information on potential implementation units between 2013 and 2017 for each of the selected countries.
- **S2**: Detailed sample size calculation.
- **S3**: An SOP on how to perform the Kato-Katz thick smear method.
- **S4**: An SOP on quality control of stool-based microscopic methods.
- **S5**: An SOP on sample preservation.
- **S6**: An SOP on DNA extraction from preserved stool samples.
- **S7**: Excel template used to estimate cost of trials in English, French and Spanish.
- **S8**: Record forms used for the trials.
- **S9**: Excel file for data entry.
- **S10**: An SOP on screening data files of two data entry clerks for mismatches and errors.
- **S11**: Summarized protocol and informed consent forms in English.
- **S12**: Summarized protocol and informed consent forms in French.
- **S13**: Summarized protocol and informed consent forms in Spanish.

Data are available under the terms of the Creative Commons Zero "No rights reserved" data waiver (CC0 1.0 Public domain dedication).
