# Supplementary material for: Piloting a surveillance system to monitor the global patterns of drug efficacy and the emergence of anthelmintic resistance in soil-transmitted helminth control programs: a Starworms study protocol
Source: Gates Open Res. 2020 Mar 10;4:28. [Version 1] doi: 10.12688/gatesopenres.13115.1 (PMC7120503; doi:10.12688/gatesopenres.13115.1)
Supplement: Peer review history

# Peer review: Reviewer response for version 1

**DOI:** 10.21956/gatesopenres.14292.r28673

**Published:** 2020-04-02

**Authors:** Taniawati Supali

**Affiliations:** 1 Department of Parasitology, Faculty of Medicine, University of Indonesia, Jakarta, Indonesia

1. In the background, the authors wrote that the study would be conducted in eight countries with high Preventive chemotherapy (PC) coverage for at least five years. It is necessary to explain the definition of “high” in PC coverage – I would say high is such a relative word, that needs more explanation regarding the number cut offs. In Table 2, the authors wrote the median national coverage of SAC was 59% in Ghana and 60% in Senegal. Are those two countries considered as high PC coverage? If Ghana and Senegal were included as countries with high PC coverage, then I assumed the “high” range of PC coverage (> 50% - 100% (wide range) was used in the study.
2. In this protocol, the authors were supposed to assess the drug efficacy of BZ drugs (Albendazole and mebendazole).  Why were there only two countries (Nicaragua and Cambodia) receiving mebendazole alone? There was also a note below Table 2 that Nicaragua has not yet confirmed for the involvement of this study. It was known as well that the efficacy of Mebendazole is different (see table 4) in Hookworms compared to Albendazole.
3. In the methodology, ethanol was needed to preserve the stool for detecting BZ resistance-associated molecular markers. Most of STH endemic areas are remote areas. It would be difficult to bring the ethanol to endemic areas by plane. Several studies had reported that FTA cards can be used to collect the stool samples for PCR. In my opinion, this method would be easier to perform in the field as well as to transport the samples from the endemic areas to the central lab.

I confirm that I have read this submission and believe that I have an appropriate level of expertise to confirm that it is of an acceptable scientific standard.

# Peer review: Reviewer response for version 1

**DOI:** 10.21956/gatesopenres.14292.r28674

**Published:** 2020-03-30

**Authors:** D. Timothy J. Littlewood

**Affiliations:** 1 Department of Life Sciences, Natural History Museum, London, UK

Addressing an important topic the study protocol is comprehensive whilst leaving some flexibility in approach towards addressing future unknowns (e.g. application of beta-tubulin SNPs). The combination of authors provides a robust overall perspective.

 I have only minor comments:

1. Although the protocol for fixing and storing stool samples for downstream molecular analysis such as qPCR is reasonably well established I would be concerned that a final concentration of 50-70% ethanol (1:1 [stool:100%EtOH] held at room temperature might not be ideal for proposed/envisaged NGS analyses.
2. I am concerned that resistance detected with a single marker gene will not in itself be efficacious, even before the proposed detection has been established as reliable. Perhaps an understanding that samples (stool/DNA) will be available for further screening studies within and beyond this study might add value to this wider investment?
3. Is the longevity of the VSSI (and samples) limited by the timespan of the investment or will this somehow be sustained beyond?
4. Similarly, publishing data on the project website suggests access will not be sustained; can these data be deposited on a recognised data portal or data site for future access?

I confirm that I have read this submission and believe that I have an appropriate level of expertise to confirm that it is of an acceptable scientific standard.
